# Burden of Mental Health among Patients with Inflammatory Bowel Disease—A Cross-Sectional Study from a Tertiary IBD Center in Hungary

**DOI:** 10.3390/jcm13072002

**Published:** 2024-03-29

**Authors:** Livia Lontai, Lívia Priyanka Elek, Fruzsina Balogh, Dorottya Angyal, Péter Pajkossy, Lorant Gonczi, Peter L. Lakatos, Ákos Iliás

**Affiliations:** 1 Department of Internal Medicine and Oncology, Semmelweis University, 1083 Budapest, Hungary; livia.lontai@gmail.com (L.L.); lorantgonczi@gmail.com (L.G.); 2 Department of Clinical Psychology, Semmelweis University, 1091 Budapest, Hungary; elek.livia.priyanka@gmail.com; 3 Faculty of Medicine, Semmelweis University, 1085 Budapest, Hungary; baloghf98@gmail.com (F.B.); adorka99@gmail.com (D.A.); 4 Department of Cognitive Science, Budapest University of Technology and Economics, 1111 Budapest, Hungary; pajkossy.peter@ttk.bme.hu; 5 Institute of Cognitive Neuroscience and Psychology, Research Centre for Natural Sciences, 1117 Budapest, Hungary; 6 Center for Cognitive Medicine, University of Szeged, 6720 Szeged, Hungary; 7 Division of Gastroenterology, McGill University Health Center, Montreal, QC H3A 0G4, Canada

**Keywords:** IBD, depression, anxiety disorder, predictive factors, cognitive behavioral therapy

## Abstract

**Background:** Inflammatory bowel diseases (IBDs) are chronic conditions that negatively affect the patient’s quality of life. With the spread of the biopsychosocial model, the role of mental health in the activity and course of inflammatory bowel disease is becoming more and more recognized. Our study aimed to assess the prevalence of anxiety and depression in IBD patients in our tertiary referral center and determine the predictive factors of these mental conditions. **Methods:** A total of 117 patients were included consecutively between 1 December 2021 and 28 February 2022. We used a questionnaire to gather demographic information, disease course, and IBD-specific symptoms. We assessed anxiety symptoms using the GAD-7 and depressive complaints using the PHQ-9 questionnaire. We evaluated disease activity using CDAI and pMayo scores. **Results:** Of the 117 patients (male/female: 63/54), 88 suffered from Crohn’s disease, and 29 were diagnosed with ulcerative colitis. Only 6 patients were taking medication for mood disorders, and 38 individuals sought mental support during their lifetime. A total of 15% of the population suffered from moderate–severe anxiety disorder, and 22% were affected by moderate–severe depression. The GAD-7 and PHQ9 values showed a significant correlation between the number of stools, bloody stools, abdominal pain, number of flare-ups, and CDAI scores. **Conclusions:** Our study confirmed that there is a high incidence of anxiety and depressive symptoms among IBD patients. Our results highlighted the symptoms that could be associated with mental disorders. It is important to assess the mental status of IBD patients to improve their quality of life.

## 1. Introduction

The incidence and prevalence of inflammatory bowel diseases (IBD), Crohn’s disease (CD), and ulcerative colitis (UC) have shown a remarkable increase in recent decades [1]. According to a previous study by our working group, nearly 50,000 patients in Hungary were affected by IBD [2]. Being a multifactorial disease, several etiological factors are known in the development of IBD (genetic, environmental, and microbiological differences in the inflammatory mechanism), but significant research is still ongoing on the subject. Inflammatory bowel disease requires lifelong treatment and a close patient–doctor relationship [3].

In the past few decades, drug treatment options have developed rapidly, and the psychological aspects have gained more and more importance in the quality of life; therefore, a more complex, biopsychosocial approach has been accepted in patient care. Chronic illness can often become a determining factor in the patient’s psychological attitudes, which in turn can affect their body image, self-esteem, and physical symptoms. It is widely recognized that psychological stress can exacerbate somatic complaints in such cases [4].

Although the importance of psychological factors in the development of IBDs has been recognized for over 60 years, the psychosomatic dichotomy was the predominant perspective on the origin of these diseases. In the current widely accepted theoretical approach, the importance of predisposing and maintaining factors is emphasized, acknowledging their involvement in the onset and progression of the illness. In the case of IBDs, stress deserves special attention, as its direct physiological impact has been confirmed in terms of changes in gastrointestinal function and bowel permeability, as well as inducing hormonal changes, altering cytokine profiles, and modifying immunological mechanisms, leading to inadequate reactions in the intestinal mucosa. Indirectly, behavioral changes related to stress can also be significant, especially in the case of health-damaging coping strategies such as smoking, which may ultimately lead to a shorter time between CD relapses [5]. Several studies have explored the impact of various stressors on the progression of the illness, distinguishing major life events from chronic stress. The increase in stressful life events can promote a flare-up of the disease, while the role of chronic, persistent stress is also significant. This underscores the importance of tailored psychological support [6,7].

Suitably chosen psychological interventions can have a beneficial effect on multiple aspects, such as the physical symptoms of the disease, the duration between relapses, and comorbid mental disorders, which are more prevalent among patients with chronic conditions, including IBD patients, compared to healthy individuals [8,9]. It is important to emphasize that the incidence of co-occurring mental health conditions is higher during the active phase of inflammatory bowel disease, but even during periods of remission, it still surpasses the rate observed in the general population [10]. It highlights the significance of disease perception, meaning the beliefs of patients regarding various aspects of the disease, including their fears of complications, the impact of symptoms on their lives, their opinions on the effectiveness of pharmacotherapy, and their evaluation of the disease’s impact on their lifestyle. The individual’s interpretation of the illness significantly contributes to the onset of comorbid depression and anxiety, and it is an important factor in the assessment of the quality of life [11].

Cognitive behavioral therapy aims to facilitate the interpretation of events and the deeper exploration of personal meaning constructions, as well as the development of realistic perceptions. This well-established psychotherapeutic approach can result in a notable reduction in symptoms of depression and anxiety in 10–12 therapeutic sessions [12]. The efficacy of interventions has been proven in coping with different somatic illnesses, including Crohn’s disease [13]. 

We aimed to assess the prevalence of anxiety and depression in patients with inflammatory bowel disease in our tertiary referral center and to determine the predictive factors of anxiety and depression observed among our patients.

## 2. Materials and Methods

In this study, we assessed the mental health of patients who attended our IBD outpatient clinic with a cross-sectional analysis. Data collection was carried out prospectively between 1 December 2021 and 28 February 2022 at the IBD center of the Department of Internal Medicine and Oncology of Semmelweis University in Hungary. Consecutive patients who visited our clinic during this period, 117 patients, could be included in the study anonymously, voluntarily, and consecutively. All patients aged ≥ 18 years and met the standard criteria of CD and UC based on the third European Evidence-based Consensus on Diagnosis and Management of Crohn’s Disease and Ulcerative Colitis [14].

In our questionnaire, we collected demographic data, followed by questions about the course of the patient’s illnesses, their current condition and medical history (number of relapses, specific symptoms, medication, surgery, and extraintestinal manifestations), and their treatment form. The disease activity was defined based on using the Crohn’s Disease Activity Index (CDAI) and the partial Mayo score (pMayo), following the recommendations of the European Crohn’s and Colitis Organisation (ECCO) guidelines. CD remission was defined as CDAI < 150, while active disease was indicated by a CDAI score > 150 [15]. As for ulcerative colitis, remission was defined as pMayo ≤ 2, while active UC was indicated by pMayo ≥ 3 [14]. We used the Generalized Anxiety Disorder Scale-7 (GAD-7) [16] to evaluate anxiety symptoms and the Patient Health Questionnaire-9 (PHQ-9) [17] to assess the occurrence of depression. Finally, we measured our patient’s psychological support by asking them if they had received professional help during their lifetime and by collecting data from National e-Health Care Cloud Hosting (EESZT) on whether they were taking any psychoactive drugs, such as anxiolytics or antidepressants.

Statistical analysis was performed using the Statistical Package for the Social Sciences software v. 20.0 (IBM Corp.; Armonk, NY, USA). For descriptive purposes, data are shown as mean (standard deviation) and *n* (%) for categorical variables. We worked with non-parametric data. We used the Mann –Whitney U test to compare two groups, and for multiple-group comparisons, we applied the Kruskal–Wallis test. We utilized the Spearman correlation coefficient to investigate associations between categorical variables. The significance level was set at *p* < 0.005 for all analyses.

## 3. Results

### 3.1. Patients Characteristics

We enrolled 117 patients in the study; 88 patients were diagnosed with Crohn’s disease, and 29 individuals suffered from ulcerative colitis. A total of 53.8% of the population were male, and 46.2% were female. The participants were between the ages of 18 and 66, with most having been diagnosed with inflammatory bowel disease between the ages of 16 and 40. 

A total of 85.2% of Crohn’s patients (*n* = 75) were in remission, while 13 patients (14%) suffered from active Crohn’s disease. In contrast, only 31% of ulcerative colitis patients (*n* = 9) were in clinical remission, while 68.9% (*n* = 20) of patients with UC exhibited mild, moderate, or severe disease activity. Three-quarters of the patients received biological therapy, of which 40 percent were prescribed with self-injection and 60 percent were administered regular infusion treatment in our outpatient clinic (injection: adalimumab or ustekinumab; infusion: infliximab or vedolizumab). 

The results obtained are summarized in Table 1.

### 3.2. Patients Mental Health

Based on responses regarding patients’ mental states, 58% of them felt balanced, while 36% reported being anxious. Unfortunately, 18 patients did not answer the PHQ9 test, but all patients responded to the GAD-7 questionnaire.

In total, 15% of the studied IBD population suffered from moderate–severe anxiety disorder, and 22% were affected by moderate–severe depression. 

In a subanalysis, we found that 19.3% of Crohn’s patients had moderate-to-severe depression, while moderate-to-severe anxiety was found in only 9% of patients with CD. Among Crohn’s disease patients in remission, 12% experienced moderate-to-severe anxiety, while 14.7% suffered moderate-to-severe depression. Of the 13 patients (14%) with active CD, 2 patients (15%) had severe anxiety, while we detected moderate or severe depression in 6 (46%) of them.

In the case of ulcerative colitis, 31 percent (*n* = 9) of them suffered from moderate-to-severe depression, while 24% (7) had moderate or severe anxiety. When we analyzed the results based on clinical activity, we found that 22.2% (*n* = 2) of UC patients in remission had moderate-to-severe anxiety, and 11.1% (*n* = 1) struggled with moderate-to-severe depression. 

In patients with clinically active UC, the incidence of moderate-to-severe anxiety was 25% (*n* = 5); however, moderate or severe depression occurred in 40% of them (*n* = 8). 

Based on the responses, out of the total 117 patients, 38 individuals (30.8%) sought psychological assistance during their lifetime. One person is seeing a mental health professional, twenty-five are attending sessions with a psychologist, nine are consulting a psychiatrist, and three are seeking help from other sources. According to the results obtained from the GAD-7 questionnaire, 41.2% of those experiencing mild anxiety, 45.5% of patients with moderate anxiety, and only 50% of severely anxious patients have ever received mental support. Regarding the PHQ-9 results, 47% of individuals with moderate depression, 40% of those with moderately severe depression, and only 33.3% of patients displaying severe depressive symptoms received psychological assistance. At the time of our survey, six patients were taking medication for mood disorders, four individuals were using anxiolytics, and two patients were being treated with antidepressants.

### 3.3. Correlations between Mental State and Clinical Symptoms

**Figure 1:**
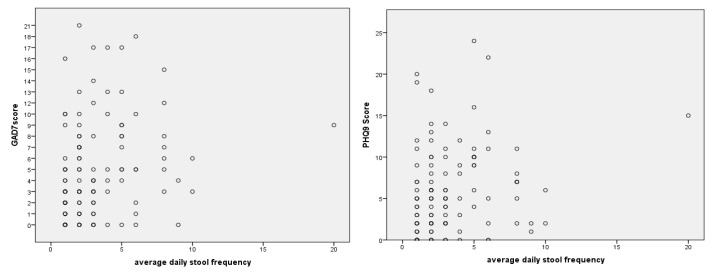
The GAD-7 and PHQ9 scores compared to the average daily stool frequency.

We conducted comparisons between the questionnaires and various clinical symptoms. There was a significant correlation between stool frequency and higher GAD-7 and PHQ9 scores (rs (115) = 0.30, *p* = 0.001, and rs (115) = 0.28, *p* = 0.002, respectively), as shown in Figure 1. Correlations remained significant even when removing the outlier (GAD-7: rs (115) = 0.29, *p* = 0.002; PHQ9: rs (115) = 0.26, *p* =0.004).

A total of 5% of the patients had bloody stools (any kind of visible blood) two weeks before filling out our questionnaires. After examining the association between bloody stools, anxiety, and depression, we found that both GAD-7 and PHQ9 scores were higher in affected patients (*p* = 0.02, *p* < 0.001). In the subanalyses, we found that bloody stools were more common in UC than in CD, but the results were not significant (*p* = 0.06). In Crohn’s patients, the number of bloody stools was associated with higher GAD-7 and PHQ9 scores (*p* = 0.02, *p* = 0.09). Patients with UC showed a significant correlation between bloody stools and depressive symptoms (*p* = 0.515), but this was not observable for anxiety symptoms. (*p* = 0.093).

Abdominal pain showed a significant correlation with the studied scores (GAD-7: χ2 (4) = 23.17, *p* < 0.001, PHQ9: χ2 (4) = 25.17, *p* < 0.001) in all IBD patients, using the Kruskall–Wallis test. However, when considering the two diseases separately, different results were observed. In UC, abdominal pain did not show a significant correlation with the results of the GAD-7 and PHQ9 questionnaires (GAD-7: χ2 (4) = 3.67, *p* = 0.453, PHQ9: χ2 (4) = 7.16, *p* = 0.127). In CD, a significant positive correlation was found between abdominal pain and both anxiety and depressive symptoms (GAD-7: χ2 (4) = 19.97, *p* = 0.001, PHQ9: χ2 (4) = 15.29, *p* = 0.004).

**Figure 2:**
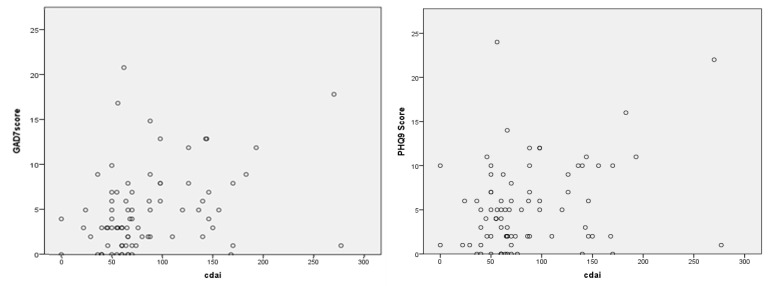
The correlation of CDAI with GAD-7 and PHQ-9 scores.

The Crohn’s Disease Activity Index (CDAI) showed a significant positive correlation with the GAD-7 and PHQ9 scores (*p* = 0.02, *p* = 0.039), as shown in Figure 2.

**Figure 3:**
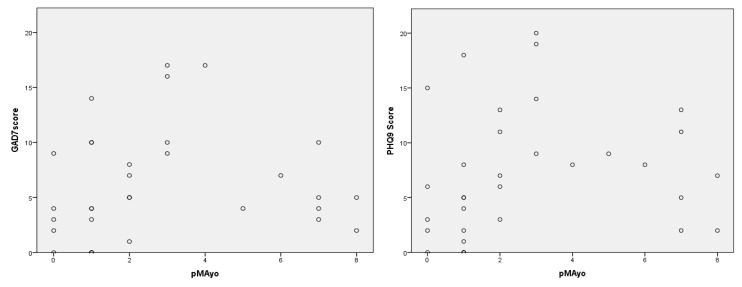
Correlations of GAD-7 and PHQ-9 scores with pMayo values.

The pMayo values showed a significant correlation with depressive symptoms (*p* = 0.034), and for anxiety disorders, there was a similar trend (*p* = 0.055). The correlations between pMayo values and GAD-7 and PHQ-9 scores are presented in Figure 3.

In 43 patients, the number of self-defined flares ranged from zero to one; in 53 patients, it ranged from two to five, while 21 patients experienced more than five relapses in the past 5 years. When comparing the number of flares with the results obtained from the GAD-7 and PHQ-9 questionnaires, we observed a significant association (GAD-7: χ2 (2) = 6.07, *p* = 0.048, PHQ9: χ2 (2) = 10.46, *p* = 0.005), indicating that the number of relapses generally has a negative impact on the patient’s anxiety and depressive symptoms.

## 4. Discussion

Our study confirmed that there is a high incidence of anxiety and depressive symptoms among IBD patients. It is important to note that, as a tertiary center, our study mainly includes patients with more severe and complex disease behaviors. Our results highlighted those simple clinical symptoms and complaints that could be associated with these mental disorders. We found in our study population that, despite the higher frequency of mental illnesses, only a small number of patients received psychological or pharmacologic help.

The optimal care of patients with ”inflammatory bowel disease poses complex challenges for healthcare professionals, and the increasing incidence of the disease puts a significant financial burden on the healthcare system. Thanks to the biopsychosocial approach, there is a growing focus on the mental well-being of these patients too. Living with a chronic illness, individuals with IBD not only have lifelong somatic complaints but also experience significant psychological difficulties and a decreased quality of life.

According to previous studies, anxiety and depression are the two most common mood disorders in this population [18,19]. In this study, we assessed the mental health of patients who attended our IBD outpatient clinic with a cross-sectional analysis. We measured patients’ anxiety using the GAD-7 questionnaire and their depressive symptoms using the PHQ-9 test. Based on the obtained scores, 15% of the 117 examined patients were found to suffer from moderate-to-severe anxiety disorders, while 22% had moderate-to-severe clinical depression. Our results are in line with the literature data. In a systematic review of 171 articles with a total of 158,371 participants, the prevalence of anxiety disorder was estimated at 20.5% and the pooled prevalence of depression was 15.2% [20].

In our study, we found that there was a higher incidence of anxiety (24%) and depression (31%) in patients with ulcerative colitis in comparison to CD patients (anxiety: 9%; depression: 19.3%). It is important to emphasize that the two groups of IBD patients differed in clinical activity. Among Crohn’s patients in remission, severe-to-moderate anxiety occurs in 12%, while depression occurs in 14.7%. In turn, 22% of ulcerative colitis patients in remission experience anxiety disorders, and 11% of them suffer from depression. In active Crohn’s disease, the prevalence of severe-to-moderate anxiety was 15%, while that of depression was 46%. In addition, 25% of patients with active ulcerative colitis had anxiety disorder, and 40% of them struggled with moderate-to-severe depression. Based on these findings, it can be concluded that among active IBD patients, the prevalence of mental disorders such as anxiety and depression is higher compared to the phase of remission. Therefore, during this period, providing appropriate psychological support to patients is particularly crucial. In contrast, we did not find a direct association between mental health outcomes and disease phenotype or exposure to biological therapy; however, patient numbers in the subgroups were small.

We compared the questionnaires with several clinical symptoms. A significant correlation was observed between higher stool frequency and both anxiety symptoms and depressive symptoms. Bloody stool also showed a significant positive correlation with the examined psychological attitudes. When split into specific diseases, it was found that while both scores showed significant results in Crohn’s disease, bloody stool did not have an impact on anxiety symptoms in ulcerative colitis and only showed a tendency but no significant correlation with depressive symptoms. These correlations can be explained by the fact that bloody stool is a common characteristic of UC, making it more accepted and less frightening for patients, whereas, in Crohn’s disease, it may indicate more severe activity and a harder-to-manage manifestation of the disease.

Assessing clinical activities, while CDAI scores showed a significant positive correlation with GAD-7 and PHQ9 results, pMayo values only demonstrated a significant association with depressive symptoms, with a positive trend observed for anxiety symptoms. This can be explained by the relatively small scale of the Mayo score in contrast with the CDAI. Based on the psychological state determined by the patients themselves, significantly lower GAD-7 and PHQ9 scores were measured among patients who provided balanced responses compared to those who felt anxious or depressed. Surprisingly, no correlation was found between the presence of fistulas and anxiety or depression symptoms among Crohn’s disease patients.

In a prospective analysis among young IBD patients, lower health-related quality of life was found to be more significantly correlated with negative illness perceptions and depression compared to demographic and disease-related factors [20]. A systematic literature review, in which forty-three studies were included, reported a significant prevalence of psychoactive drug utilization among IBD patients, but only a small fraction received psychiatric referrals. Approximately one-third of the studies indicated that psychotherapy had a positive impact on enhancing the quality of life, stress perception, and levels of anxiety and depression in individuals with inflammatory bowel disease. Furthermore, antidepressants proved effective in reducing disease activity and gastrointestinal symptoms as well [21].

According to international guidelines, including those of the European Crohn-Colitis Organization, it is recommended to monitor comorbid mental disorders not only among patients with active IBD but also during remission. If necessary, professional help should be applied. It is presumed that psychotherapy not only alleviates anxiety and depressive symptoms but also has a favorable impact on gastrointestinal complaints and disease activity [22,23]. Among psychotherapies, cognitive behavioral therapy has been proven to reduce anxiety and depressive symptoms in medical practice. It aims to identify and modify negative automatic thoughts, dysfunctional attitudes, maladaptive schemas, and cognitive distortions, instead of developing a more adaptive interpretation of the situation. Currently, there is still limited evidence-based literature available on the effectiveness of cognitive behavioral therapy in IBD patients. Based on existing findings, it can be concluded that its use can be a personalized therapeutic approach in the multidisciplinary management of inflammatory bowel disease.

The strength of our study is that we examined a consecutive population with severe disease courses from our tertiary referral IBD center. We used generally accepted and internationally approved psychological scores. We explored clinical disease activity, psychological symptoms, and the presence of psychological help (mood-enhancing drugs and mental support) at the same time. After summarizing the results, we singled out those suffering from serious mental illness and provided them with psychological support.

The limitation of this paper is the low sample size, which likely distorts our calculations, and it could be the underlying reason for the biased results obtained for patients with ulcerative colitis. With our findings, we have demonstrated that there is a high prevalence of anxiety disorders and depressive symptoms among IBD patients. It is important to note that the study population consists of patients with complex phenotypes, a high extraintestinal manifestation rate, and a severe disease course. This not only leads to a deterioration in quality of life but also poses significant compliance issues, which can impair the effectiveness of a well-chosen therapy.

## 5. Conclusions

In conclusion, it can be stated that there is a high prevalence of mental comorbidities among patients with inflammatory bowel disease. During their care, especially during periods of active disease, they require special attention in terms of appropriate psychological support, and it is important to screen and treat mood disorders to achieve long-term therapeutic success and improve patients’ quality of life. Our study has identified the symptoms that can be risk factors for anxiety disorders and depression. In case of their occurrence, it may be necessary to provide psychological support as part of the complex management of inflammatory bowel diseases.

Based on the results obtained, we implement psychological support for IBD patients treated in our tertiary referral center by offering psychological consultations. They have the opportunity to participate in individual therapy. In addition, we started an eight-session cognitive behavioral therapy group specifically for patients suffering from inflammatory bowel disease. Our goal was to develop an adaptive therapy for the treatment of IBD patients in Hungary, promoting more professional psychological care for these patients.

In addition, we intend to integrate periodic evaluations of the mental status of patients with inflammatory bowel disease as part of comprehensive care to identify patients with mental difficulties and provide them with appropriate psychological support.

## Figures and Tables

**Table 1:** Characteristics of the study population (*n* = 117).

Demographical Data	
Type of IBD (CD, UC)	88 (75%), 29 (25%)
Age (average, years) (±SD)	36.5 (±12.13)
Age at diagnosis (±SD)	23.9 (±10.49)
Montreal classification A1/A2/A3	25, 81, 11 (21.4%, 69.2%, 9.4%)
Gender distribution: male, female	63 (53.8%), 54 (46.2%)
Marital status: single, in relationship, married/life partner, divorced	28 (23.9%), 27 (23.1%), 58 (49.6%), 4 (3.4%)
Education: elementary school, high school, training, college, university	3 (2.6%), 39 (33.3%), 30 (25.6%), 19 (16.2%), 26 (22.2%)
**Clinical Activity**	
Number of relapses in the last 5 years: 0–1, 2–5, more than 5	43 (36.8%), 53 (45.3%), 21(17.9%)
Active disease (patient’s self-opinion): yes, no	46 (39.1%), 71 (60.9%)
Stool frequency per day: 1, 2, 3, 4, 5, 6, 8, 9, 10, 20	32 (27.6%), 29 (25%), 18 (15.5%), 7(6%), 11 (9.4%), 7 (6%), 7 (6%), 2 (1.7%), 2 (1.7%), 1 (0.9%)
Bloody stool in the last 2 weeks: yes, no	22 (18.8%), 95 (81.2%)
Frequency of abdominal pain: daily, 2–3×/week, weekly, monthly, less often	15 (12.8%), 11 (9.4%), 10 (8.5%), 22 (18.8%), 59 (50.4%)
Fistula: yes, no	28 (24%), 89 (76%)
Patients in remission/ active disease (based on CDAI and pMAyo) CDAI, pMayo (average) (±SD)	CD 75 (85.2%)/ 13 (14.8%), UC 9 (31%)/ 20 (69%) 89 (±60.8), 3 (±2)
**Treatment**	
Conservative therapy: topical, systemic	30 (25.6%) 1 (0.8%), 29 (24.8%)
Biological therapy: injection, infusion	87 (74.4%) 35 (29.9%), 52 (44.4%)
IBD-related operation: yes, no	23 (19.7%), 94 (80.3%)
Having a stoma: yes, no, currently	9 (7.7%), 107 (91.4%), 1 (0.85%)
**Mental State**	
Current state of mind (patient’s self-opinion): balanced, worried/anxious, depressed, desperate	68 (58.1%), 42 (35.9%), 4 (3.4%), 3 (2.6%)
GAD-7 results: minimal, mild, moderate, severe anxiety	65 (55.5%), 34 (29%), 11 (9.4%), 7 (5.9%)
PHQ9 results: minimal, mild, moderate, moderately severe, severe depression	37 (37%), 36 (36%), 19 (19%), 4 (4%), 3 (3%)

## Data Availability

The data are not available for public access because of patient privacy concerns but are available from the corresponding author upon reasonable request.
